# Unexpected Air on Trauma Computed Tomography: Iatrogenic Intravascular Air After Peripheral Venous Cannulation

**DOI:** 10.7759/cureus.107265

**Published:** 2026-04-17

**Authors:** Takao Zama, Tatsuya Tanaka, Takashi Furukawa, Taku Goto, Hiroko Nakashiro, Yukinori Takase, Eiichi Suehiro, Akira Matsuno

**Affiliations:** 1 Department of Neurosurgery, International University of Health and Welfare Narita Hospital, Narita, JPN; 2 Department of Neurosurgery, Kouhou-kai Takagi Hospital, Okawa, JPN; 3 Department of Emergency Medicine, Kouhou-kai Takagi Hospital, Okawa, JPN; 4 Department of Emergency Medicine, International University of Health and Welfare Narita Hospital, Narita, JPN

**Keywords:** central venous air, computed tomography, differential diagnosis, elderly trauma, iatrogenic complication, incidental findings, intravascular air, peripheral venous cannulation, trauma ct, venous air embolism

## Abstract

Small amounts of intravascular air are occasionally detected on computed tomography (CT), most commonly after intravenous contrast administration. However, intravascular air may also be introduced during routine peripheral venous access before contrast injection. In the trauma setting, this finding may be misinterpreted as traumatic mediastinal or thoracic pathology, particularly in elderly patients. We report the case of an 89-year-old man who presented after a ground-level fall with mild head trauma. A 20-gauge peripheral intravenous catheter was inserted into the left median cubital vein before imaging. Non-contrast head CT demonstrated traumatic subarachnoid hemorrhage. Cervical CT incidentally revealed a small focus of air within the left subclavian vein, and subsequent chest CT demonstrated a new small focus of air within the superior vena cava. Follow-up imaging showed complete disappearance of the air. No pneumomediastinum, pneumothorax, airway injury, esophageal abnormality, or other thoracic traumatic pathology was identified. The patient remained asymptomatic throughout the clinical course and developed no signs of symptomatic air embolism. The anatomical distribution, temporal migration, and spontaneous disappearance of the air suggested a benign iatrogenic origin related to peripheral venous access rather than traumatic pathology or contrast administration. This case highlights the importance of recognizing incidental central venous air as a potential imaging pitfall in elderly trauma patients in order to avoid unnecessary diagnostic workup and overinterpretation.

## Introduction

Computed tomography (CT) is commonly used in the initial evaluation of elderly trauma patients to exclude intracranial hemorrhage, cervical spine injury, and other clinically important complications [1,2]. In daily practice, such imaging may also reveal incidental findings unrelated to the traumatic event itself [3-5]. One such finding is the presence of small amounts of intravascular or intracardiac air [3-5].

Venous air embolism is a well-recognized incidental finding on contrast-enhanced CT [3,4,6,7]. However, it is less widely appreciated that intravascular air may also be introduced during routine peripheral venous cannulation or line manipulation, even before contrast administration [4,5]. Previous studies have shown that small amounts of intravascular air are not uncommon on CT, with reported incidences ranging from approximately 11% to over 30%, and are most often located in the right-sided cardiac chambers or central veins [3-5].

Although these air bubbles are usually asymptomatic and clinically insignificant, their detection on trauma CT can be alarming [3,4,6]. In elderly trauma patients in particular, the presence of air around the mediastinum or heart may raise concern for serious traumatic conditions such as tracheobronchial injury, esophageal perforation, or traumatic mediastinal emphysema. Misinterpretation of benign iatrogenic intravascular air as a traumatic or pathological finding may lead to unnecessary additional investigations and overdiagnosis.

Therefore, it is important for clinicians and radiologists to recognize the characteristic distribution and clinical context of incidental intravascular air associated with peripheral venous access [3-5]. We present a case of an elderly trauma patient in whom left-sided intravascular air was incidentally detected on pre-contrast CT after routine peripheral venous cannulation. This finding may represent a diagnostic pitfall, as the presence of air on pre-contrast imaging can be misinterpreted as traumatic mediastinal pathology, such as pneumomediastinum. This case highlights the importance of distinguishing iatrogenic intravascular air from traumatic pathology during CT interpretation in the acute trauma setting.

## Case presentation

An 89-year-old man, who had been independent in activities of daily living, was brought to our hospital after sustaining a head injury from a ground-level fall. His medical history included bladder cancer, prostate cancer, and age-related macular degeneration. He did not consume alcohol at the time of presentation and had quit smoking approximately 30 years earlier.

On arrival, he was transported with a cervical collar for spinal immobilization. His vital signs were as follows: blood pressure 202/99 mmHg, heart rate 82 beats/min, respiratory rate 18 breaths/min, body temperature 36.8°C, and oxygen saturation 100% on room air. He was alert, with a Glasgow Coma Scale score of 15 [8], and had no disturbance of consciousness, amnesia, nausea, vomiting, neck pain, chest pain, dyspnea, or dysphagia.

Physical examination revealed an approximately 3-cm laceration over the right temporo-occipital scalp. No focal neurological deficits were observed.

**Figure 1:**
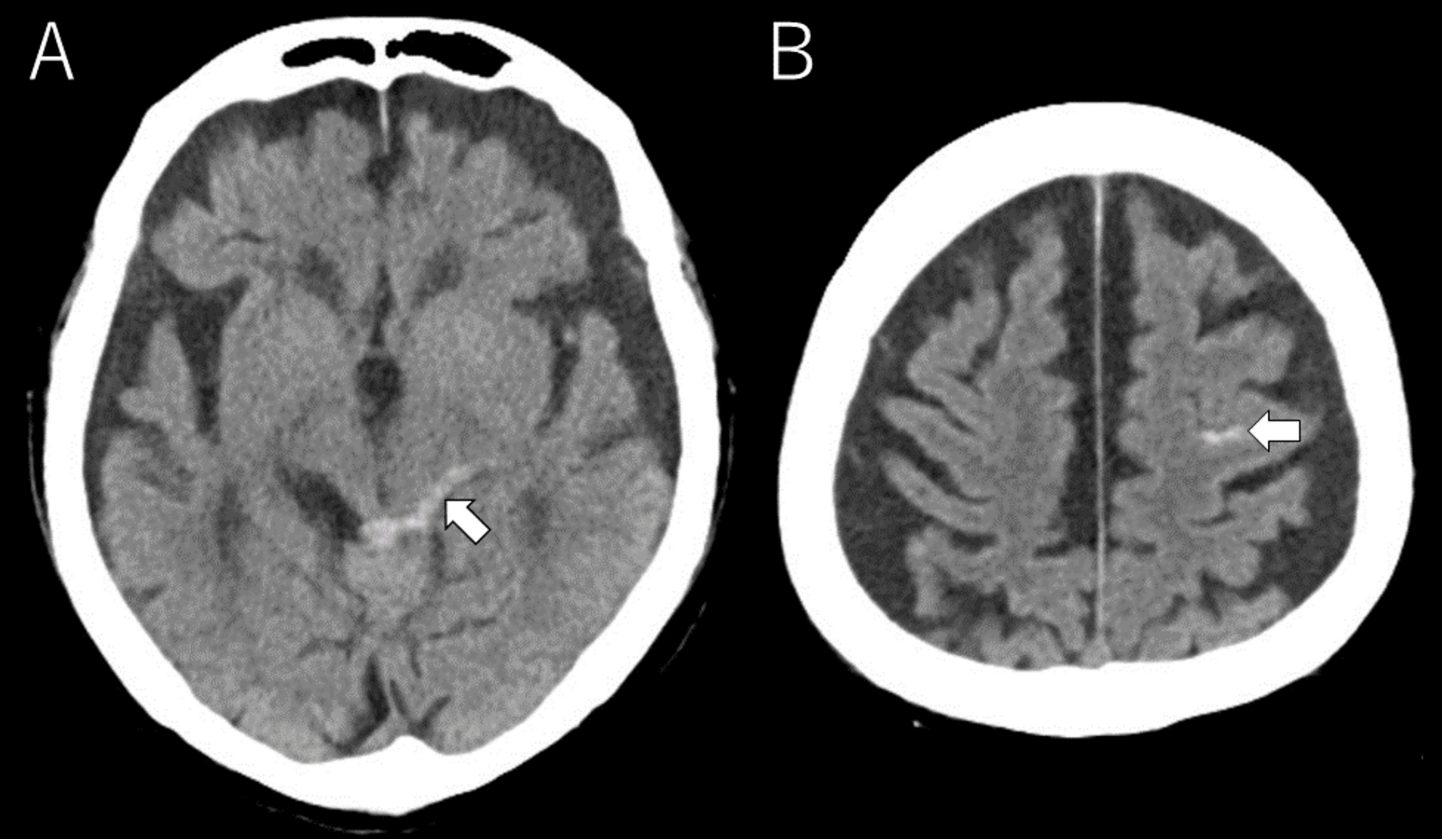
Initial head CT showing traumatic subarachnoid hemorrhage. (A, B) Axial non-contrast head CT images showing traumatic subarachnoid hemorrhage (arrows).

At 9:35 a.m., a 20-gauge peripheral intravenous catheter was inserted into the left median cubital vein. After insertion, blood sampling was performed, and the line was connected and secured with confirmation of adequate drip infusion. No saline flushing was performed prior to imaging. Subsequently, head CT and cervical CT were performed at 9:44 a.m. Non-contrast head CT demonstrated traumatic subarachnoid hemorrhage in the left frontal sulcus and around the left ambient/quadrigeminal cistern (Figure 1). No skull fracture was identified.

**Figure 2:**
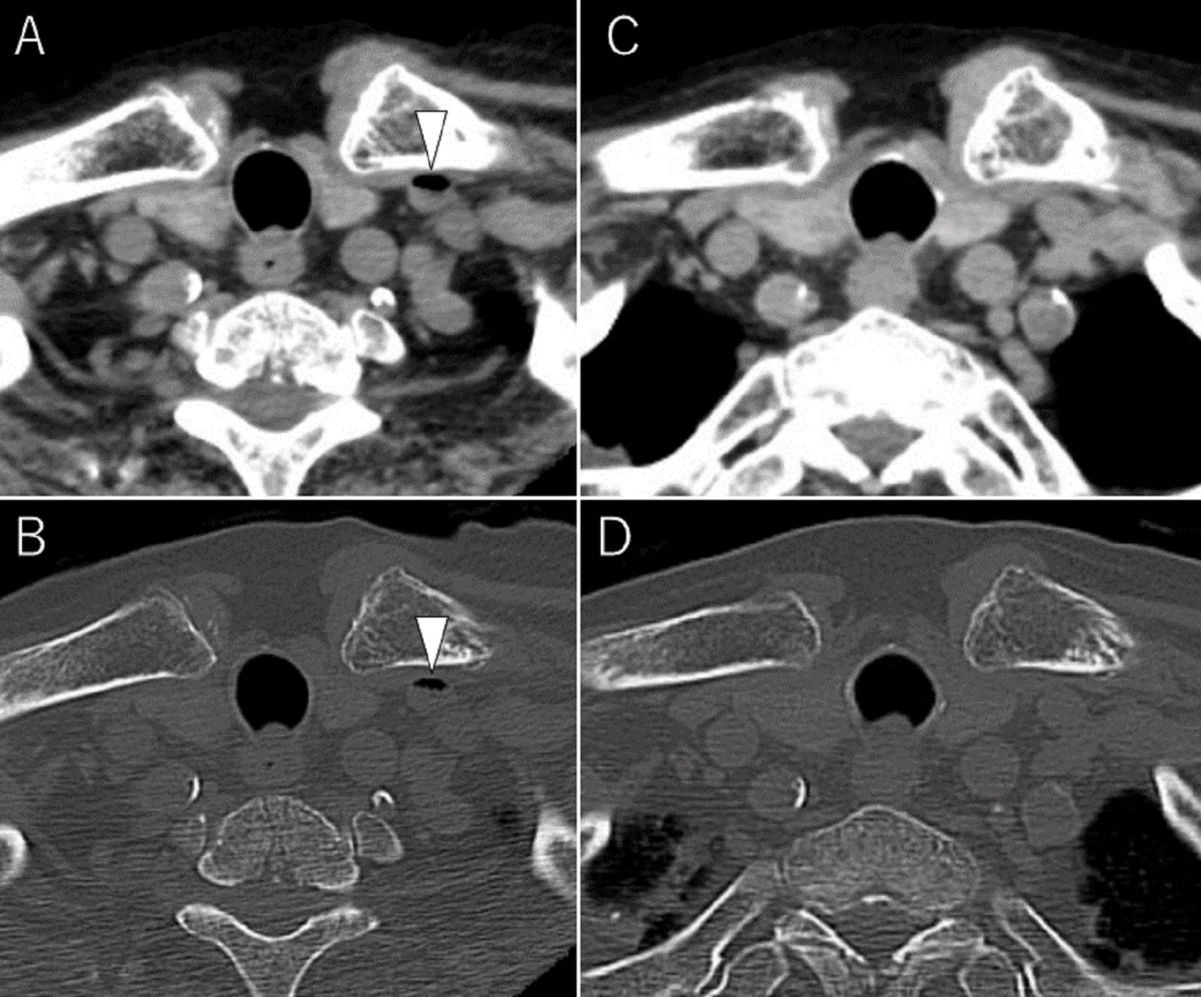
Air in the left subclavian vein on cervical CT. (A) Axial cervical CT image (soft tissue window) showing a small focus of air within the left subclavian vein (arrowhead). (B) Corresponding bone window image confirming the same finding (arrowhead). (C, D) Follow-up CT images at the same level, showing the disappearance of air from the left subclavian vein.

Cervical CT performed at the same time showed no cervical fracture or dislocation. However, a small focus of air was incidentally detected within the left subclavian vein (Figure 2A, 2B). On follow-up CT at the same anatomical level, the air within the left subclavian vein was no longer visible (Figure 2C, 2D).

**Figure 3:**
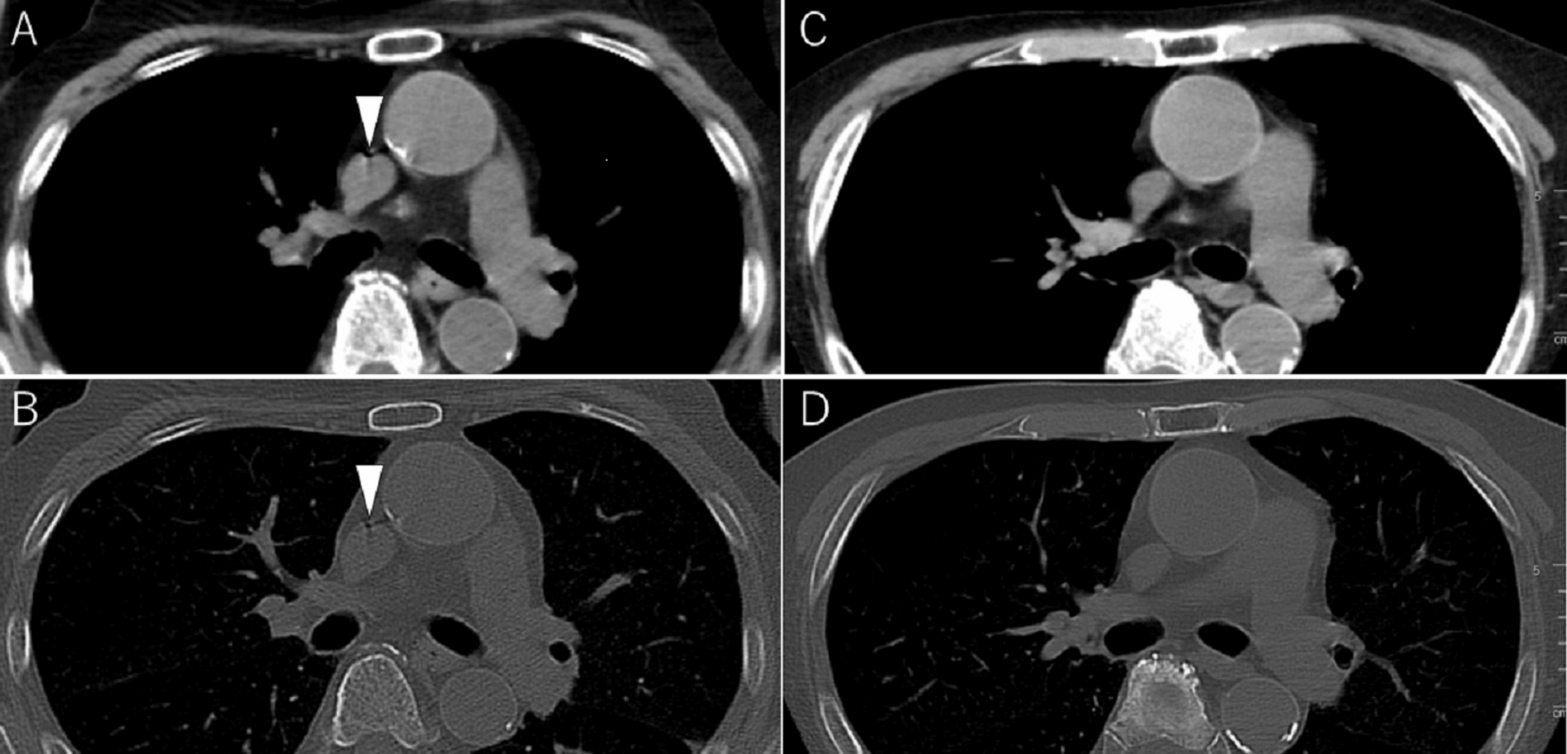
Air in the superior vena cava on subsequent chest CT and its later disappearance. (A) Axial chest CT image (soft tissue window) showing a small focus of air within the superior vena cava (arrowhead). (B) Corresponding bone window image confirming the same finding (arrowhead). (C, D) Follow-up CT images at the same level, showing later disappearance of the air.

A chest and abdominal CT obtained at 9:50 a.m. demonstrated a new small focus of air within the superior vena cava (Figure 3A, 3B). At that time, there was no evidence of pneumomediastinum, pneumothorax, pleural effusion, periesophageal abnormality, or airway injury. A follow-up head and neck CT performed at 3:25 p.m. demonstrated complete disappearance of the superior vena cava air (Figure 3C, 3D).

The traumatic subarachnoid hemorrhage was managed conservatively with tranexamic acid and lacosamide. Throughout the clinical course, the patient remained neurologically stable and developed no chest symptoms, respiratory symptoms, dysphagia, or other clinical manifestations suggestive of symptomatic air embolism.

## Discussion

In the acute evaluation of elderly trauma patients, computed tomography (CT), often including head and cervical spine imaging, is widely used to avoid overlooking clinically significant injuries, even after minor trauma [1,2,9,10]. Guidelines and national data in Japan emphasize the importance of early imaging-based assessment, particularly because geriatric traumatic brain injury may initially present with mild findings despite a risk of deterioration [1,2,9,10].

During such imaging evaluation, unexpected findings unrelated to the primary traumatic lesion may occasionally be encountered. In the present case, central venous air was identified as such an incidental finding. Because the air was already visible on pre-contrast CT, it was considered more likely to be iatrogenic intravascular air associated with peripheral venous cannulation, line connection, or saline flushing, rather than contrast injection itself.

Although venous air embolism is classically recognized as an incidental finding on contrast-enhanced CT, more recent studies have shown that intravascular air can also be detected on non-contrast CT performed after intravenous cannulation [3,4]. Groell et al. reported intravascular air in 79 of 677 patients (11.7%) undergoing contrast-enhanced chest CT and further demonstrated that air was already present on unenhanced CT in a subset of patients, indicating that air entry may occur before contrast administration [3]. More recently, Samji et al. showed that intravascular air was detected in 27 of 110 patients (24.5%) on non-enhanced CT after IV cannulation and in 36 of 110 patients (32.7%) after contrast administration, whereas no air was identified in a control group without intravenous cannulation [4]. These findings strongly suggest that air may be introduced not only by power injection of contrast material but also during the earlier steps of routine venous access and line handling, which is entirely consistent with the mechanism suspected in the present case.

The distribution of intravascular air also provides an important clue to its origin. Venous air introduced through a peripheral intravenous line is most commonly observed in the right atrium, right ventricle, superior vena cava, brachiocephalic vein, or main pulmonary artery, reflecting the normal course of venous return [3,4,7,11]. In the study by Groell et al., the main pulmonary artery was the most frequent site, followed by the superior vena cava, right ventricle, subclavian/brachiocephalic veins, and right atrium [3]. Samji et al. similarly found that the majority of intravascular air on non-enhanced CT was located in the right atrial appendage/right atrium [4]. Therefore, air limited to the right heart and central venous system, as in the present case, is anatomically typical of benign iatrogenic venous air.

In elderly trauma patients, the presence of air around the mediastinum or heart requires careful differentiation between intravascular air and traumatic extravascular air. Intravascular air is typically confined to the venous system, including the right heart and central veins, and follows the normal course of venous return. In contrast, traumatic extravascular air is usually distributed outside the vascular structures and is often associated with findings such as subcutaneous emphysema, pneumothorax, mediastinal fluid collection, or direct evidence of airway or esophageal injury. In addition, intravascular air may demonstrate gravity-dependent distribution or form air-fluid levels within the cardiac chambers, reflecting its intraluminal nature, which further helps distinguish it from extravascular mediastinal air. This distinction is clinically important to avoid misinterpretation and unnecessary investigations.

Nevertheless, intravascular air should not be regarded as universally trivial. Although small volumes are usually asymptomatic and often resolve spontaneously, larger amounts of air or the presence of a right-to-left shunt, such as a patent foramen ovale, may lead to serious complications, including paradoxical cerebral or coronary embolism [4,7,11,12]. Sodhi et al. emphasized that larger volumes of venous air can become hemodynamically significant, and prior reports have described potentially fatal outcomes depending on the volume and route of air entry [11]. Accordingly, the finding of intravascular air should be interpreted in light of the patient’s overall condition, cardiopulmonary status, and risk factors for paradoxical embolization.

## Conclusions

This case highlights that central venous air incidentally detected during CT evaluation of an elderly trauma patient may be related to peripheral venous cannulation rather than contrast administration. When air is confined to the right heart or central venous system, clinicians should consider the possibility of iatrogenic intravascular air while carefully differentiating it from traumatic pathology.

## References

[1] (2019). Guidelines for the Management of Head Injury. 4th ed. [In Japanese]. Guidelines for the Management of Head Injury. 4th ed. [in Japanese].

[2] (2021). Guidelines for Initial Trauma Treatment, Japan Advanced Trauma Evaluation and Care (JATEC), 6th ed. [In Japanese]. Japan Advanced Trauma Evaluation and Care (JATEC). 6th ed. [in Japanese].

[3] Groell R, Schaffler GJ, Rienmueller R, Kern R (1997). Vascular air embolism: location, frequency, and cause on electron-beam CT studies of the chest. Radiology.

[4] Samji KB, Chandrarathne GS, Khan W, Jones H, Owen R, Vethanayagam D (2025). Contrast-enhanced cardiac computed tomography and the presence of intravascular air: a patient safety study. J Clin Med.

[5] Tanabe R, Matsuura H, Otsuka Y, Endo A (2020). Dynamic Mercedes-Benz sign in the right atrium. JMA J.

[6] Woodring JH, Fried AM (1988). Nonfatal venous air embolism after contrast-enhanced CT. Radiology.

[7] Ie SR, Rozans MH, Szerlip HM (1999). Air embolism after intravenous injection of contrast material. South Med J.

[8] Teasdale G, Jennett B (1974). Assessment of coma and impaired consciousness. A practical scale. Lancet.

[9] Suehiro E, Fujiyama Y, Kiyohira M, Haji K, Ishihara H, Nomura S, Suzuki M (2019). Risk of deterioration of geriatric traumatic brain injury in patients treated with antithrombotic drugs. World Neurosurg.

[10] Suehiro E, Tanaka T, Michiwaki Y (2023). Fact-finding survey of treatment of traumatic brain injury in Japan: standardization of care and collaboration between neurosurgery and emergency departments. World Neurosurg.

[11] Sodhi KS, Das PJ, Malhotra P, Khandelwal N (2012). Venous air embolism after intravenous contrast administration for computed tomography. J Emerg Med.

[12] Kayano S, Takahashi A, Ota H, Takase K (2019). A case of iatrogenic air bubbles in the left ventricle by coronary computed tomographic angiography. Radiol Case Rep.

